# P-1166. Obeldesivir clinical dose projection for Marburg virus infection post-exposure prophylaxis

**DOI:** 10.1093/ofid/ofaf695.1359

**Published:** 2026-01-11

**Authors:** Jack Chang, Eric Salgado, Darius Babusis, Meghan Vermillion, Santosh Davies, Roy Bannister, Yoshihiko Murata, Luzelena Caro

**Affiliations:** Gilead Sciences, Inc., Foster City, CA; Gilead Sciences, Inc., Foster City, CA; Gilead Sciences, Inc., Foster City, CA; Lovelace Biomedical Research Institute, Albuquerque, New Mexico; Gilead Sciences, Inc., Foster City, CA; Gilead Sciences, Inc., Foster City, CA; Gilead Sciences, Inc., Foster City, CA; Gilead Sciences, Inc., Foster City, CA

## Abstract

**Background:**

Marburg virus disease (MVD) is a rapidly progressive hemorrhagic disease with fatality rates of up to 90% that currently lacks an approved vaccine or treatment. Obeldesivir (ODV) is an investigational agent with antiviral activity against Marburg virus (MARV) and efficacy as a post-exposure prophylaxis (PEP) agent against lethal MARV challenge in nonhuman primates (NHP). A clinical dose projection was conducted to balance safety and efficacy, aiming to optimize therapeutic outcomes while minimizing potential for adverse effects.

**Methods:**

Safety and pharmacokinetic (PK) data from prior ODV clinical trials were used to develop a plasma GS-441524 population PK model for the ODV metabolite GS-441524 and to select the target ODV dose for MARV PEP. A logistic regression PK/pharmacodynamic model was developed to characterize the relationship between plasma exposure and creatinine clearance (CrCL) treatment-emergent laboratory abnormalities (TELAs). This model was utilized to predict the percentage of participants with CrCL TELAs across various ODV dosing regimen scenarios. The clinical PK efficacy target for MARV PEP was based on NHP plasma exposures of GS-441524 associated with NHP efficacy from a PK study in uninfected NHP. The anticipated 24-hour exposure (AUC_0-24h_) following a 100 mg/kg/day regimen in NHP was selected as the PK efficacy target on Day 1 when evaluating different clinical ODV dosing regimens. PK simulations were then performed to evaluate dosing regimens that would achieve comparable exposures in adults ( >18 years) with normal renal function (eGFR ≥90 mL/min/1.73 m^2^) and those with mild renal impairment (eGFR 60-89 mL/min/1.73 m^2^).

**Results:**

Based on simulations for participants with normal renal function and mild renal impairment, a clinical dosing regimen was selected to meet or exceed the NHP PK efficacy target in >75% of the population on Day 1 with an acceptable rate of CrCL TELAs (dose to be presented on poster).

**Conclusion:**

This modeling and simulation approach allowed an informed dose selection to ensure clinical plasma exposures of GS-441524 will meet or exceed the NHP efficacy target with an acceptable predicted percentage of CrCL TELAs in participants with eGFR ≥60 mL/min/1.73 m^2^. PK and safety of the projected ODV dose will be assessed in clinical trials.

**Disclosures:**

Jack Chang, Jr., PharmD, MSCI, Gilead Sciences, Inc.: Employee|Gilead Sciences, Inc.: Stocks/Bonds (Public Company) Eric Salgado, PhD, Gilead Sciences, Inc.: Employee|Gilead Sciences, Inc.: Stocks/Bonds (Public Company) Darius Babusis, BA, Gilead Sciences, Inc.: Employee|Gilead Sciences, Inc.: Stocks/Bonds (Public Company) Meghan Vermillion, DVM, PhD, Gilead Sciences, Inc.: Employee|Gilead Sciences, Inc.: Stocks/Bonds (Public Company) Santosh Davies, MD, Gilead Sciences, Inc.: Employee|Gilead Sciences, Inc.: Stocks/Bonds (Public Company) Roy Bannister, PhD, Gilead Sciences, Inc.: Employee|Gilead Sciences, Inc.: Stocks/Bonds (Public Company) Yoshihiko Murata, MD, PhD, Gilead Sciences, Inc.: Employee|Gilead Sciences, Inc.: Stocks/Bonds (Public Company) Luzelena Caro, PhD, Gilead Sciences, Inc.: Employee|Gilead Sciences, Inc.: Stocks/Bonds (Public Company)

